# Post-COVID-19 Syndrome as Described by Patients: A Qualitative Study

**DOI:** 10.3390/healthcare13070757

**Published:** 2025-03-28

**Authors:** Federico Fonda, Stefania Chiappinotto, Erica Visintini, Denise D’Elia, Terence Ngwache, Maddalena Peghin, Carlo Tascini, Matteo Balestrieri, Marco Colizzi, Alvisa Palese

**Affiliations:** 1 School of Nursing, Department of Medicine (DMED), University of Udine, 33100 Udine, Italy; federico.fonda@uniud.it (F.F.); stefania.chiappinotto@uniud.it (S.C.); erica.visintini@asufc.sanita.fvg.it (E.V.); 2 Infectious Diseases Division, Department of Medicine (DMED), University of Udine, 33100 Udine, Italy; denisedelia@outlook.it (D.D.); ngwa_che@yahoo.com (T.N.); carlo.tascini@uniud.it (C.T.); 3 Infectious and Tropical Diseases Unit, Department of Medicine and Surgery, University of Insubria-ASST-Sette Laghi, 33100 Varese, Italy; maddalena.peghin@gmail.com; 4 Unit of Psychiatry, Department of Medicine (DMED), University of Udine, 33100 Udine, Italy; matteo.balestrieri@uniud.it

**Keywords:** COVID-19, SARS-CoV-2, post-acute COVID-19 syndrome, qualitative research

## Abstract

**Background/Objectives**: Growing interest in post-viral conditions following COVID-19 infection has led researchers and clinicians to develop several definitions of post-COVID-19 syndrome. This study aimed to understand the meaning given to post-COVID-19 syndrome by individuals who survived the first wave of the pandemic two years after its onset. **Methods**: A descriptive qualitative study was performed according to the Standards for Reporting Qualitative Research guidelines. An inductive and content analysis were adopted on narratives collected via the interview of patients who had been infected with SARS-CoV-2 during the first pandemic wave in the Friuli Venezia Giulia Region (Italy). **Results**: This study included 230 patients, of whom 158 experienced post-COVID-19 syndrome, and 46 (29.1%) reported suffering from this condition 24 months after the infection. On average, patients experienced three symptoms, with most of them experiencing at least one. Seventy-five patients reported being familiar with the definition of the post-COVID-19 syndrome, mainly through media and the internet (28.9% and 28.2%, respectively). The post-COVID-19 syndrome was described as characterized by two themes: (a) the experience of interrelated physical and psychological symptoms and (b) the experience of fighting like warriors for a long time. **Conclusions**: The post-COVID-19 syndrome is highly prevalent but poorly understood. Patients rely on low-quality information rather than that offered by clinicians. The post-COVID-19 syndrome appears to be a complex syndrome encompassing physical and mental symptoms, as well as those disabling the person with an unclear trajectory. There is a need to focus on the long-term consequences of COVID-19, incorporating insights from individuals’ lived experiences.

## 1. Introduction

The Coronavirus Disease 2019 (COVID-19) pandemic, caused by the Severe Respiratory Syndrome Coronavirus 2 (SARS-CoV-2), has profoundly impacted the world at multiple levels, affecting individuals and whole societies. The World Health Organization (WHO) declared COVID-19 as a Public Health Emergency of International Concern (PHEIC) in January 2020 [1] and, two months later, as a pandemic [1,2]. From its very beginning, governments and public health authorities tried to face the health emergency, establishing several health measures to prevent large-scale disease transmission [3], while researchers focused on COVID-19 prevention, diagnosis, treatment, and prognosis. The extraordinary research efforts led to the development of effective vaccines that significantly reduced severe cases, deaths, and hospitalizations [4].

After initial research efforts centered on acute COVID-19 management, bibliometric analyses showed a shifting trend in investigations [5], with growing interest directed towards post-viral conditions, a domain that had been relatively neglected before [6]. The long-term sequelae of COVID-19, including persistent symptoms and a wide spectrum of clinical manifestations, started to be closely investigated, with attempts to provide diagnostic criteria to classify the phenomenon that resulted in multiple definitions from different health authorities (Supplementary Table S1). Commonly recognized definitions included terms such as post-COVID-19 syndrome, post-acute COVID-19 syndrome, chronic COVID-19, long-term effects of COVID-19, long COVID-19, and post-acute sequelae of SARS-CoV-2 infection [7]. A state-of-the-art review of post-COVID-19 syndrome [7] found that Greenhalgh et al. in 2020 [8] first defined post-acute-COVID-19 as an illness extending beyond three weeks after the acute onset of symptoms [7,8]. Since then, research priority was progressively given to addressing and managing the burden of the so-called post-COVID-19 syndrome on patients over time [7]; epidemiological data have also indicated the high prevalence of healthcare services utilization after the COVID-19 infection, with up to 40% of patients requiring contact with the healthcare system, 18% of patients receiving a new medical diagnosis after COVID-19, of which 35% were associated with the infection by the physician [9].

Accumulated evidence indicates that COVID-19 disease is multifaceted and complex, with multiple clinical manifestations—both somatic and psychological—that can persist long after the infection [10]. On the one hand, conceptual definitions of the phenomenon by authorities and scientific institutions provide a shared framework for clinicians that tends to focus on observable behaviors, symptoms or implications, with standardized measures and languages. On the other hand, patients provide different descriptions of their lived experiences, generally reflecting a negative impact of COVID-19 on their daily lives [11], supporting the need of tailored healthcare pathways [11]. There is thus a need to reconcile the “etic” (outsider) and “emic” (insider) perspectives [12,13], by carrying patient-centered research initiatives aimed at discovering the post-COVID-19 syndrome as experienced from the point of view of people, especially involving those who have been firstly infected as their long-term experience was firstly lived.

Italy was one of the first Western countries to be heavily affected by COVID-19 [14], with dramatic social consequences [15]. Available studies investigating the post-COVID-19 syndrome among Italian patients have reported high psychological distress, including anxiety and depression [16]. In a qualitative phenomenological study on 17 women, after the COVID-19 infection, a radical change in their lives, feelings of uncertainty, and significant psycho-emotional effects have been reported [17]. Continuing to investigate how individuals from the first wave perceive living with post-COVID-19 syndrome and the meaning they attribute to their experiences is imperative because it can enhance our understanding from the emic perspective [12], valuing the so-called Patient-Reported Experience. Furthermore, it may help in deepening the impact of this condition when examined in relation to its prevalence within a cohort. Overall, this study aimed at investigating the emic perspective of post-COVID-19 syndrome among a cohort of COVID-19 survivors of the first pandemic wave (March 2020), two years after the infection onset.

## 2. Materials and Methods

### 2.1. Study Design

This qualitative descriptive study [18,19] was nested within a larger prospective monocentric cohort study named CORonavirus MOnitoRing part 4 (CORMOR 4) [10], which investigated antibody responses among individuals infected with SARS-CoV-2. An inductive approach and a content analysis were adopted [20,21] to analyze patients’ narratives [22] regarding their underlying complex and multifaceted dimensions of post-COVID-19 experience [23]. Standards for Reporting Qualitative Research (SRQR) guidelines were followed [24], as described in Supplementary Table S2.

### 2.2. Setting and Participants

**Figure 1:**
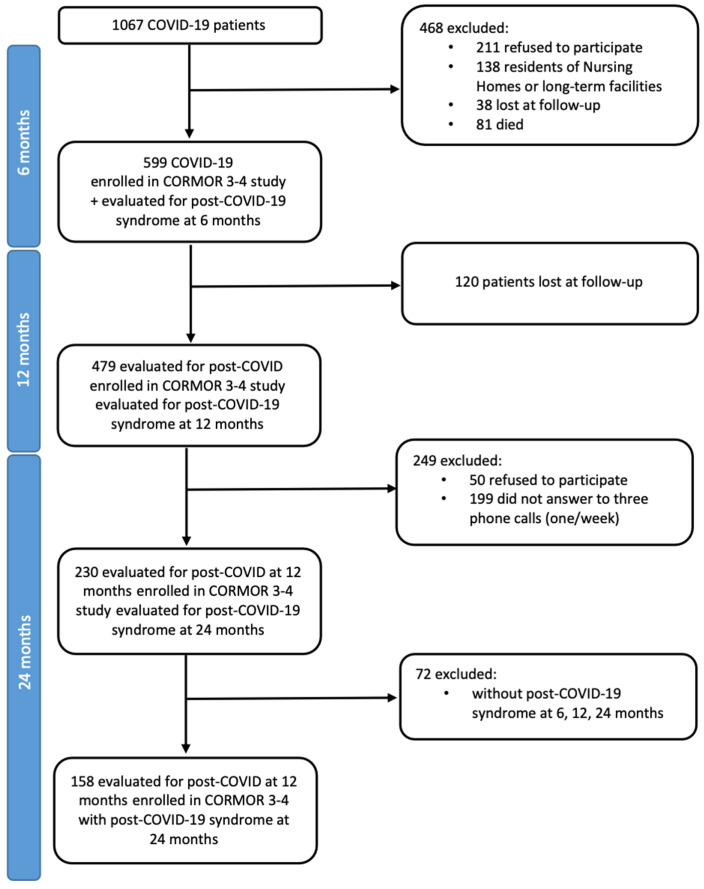
Flow diagram of patients with COVID-19 eligible and included in the CORMOR3-4 study and those included in this qualitative study design. Legend. COVID-19, Coronavirus Disease 2019; CORMOR 3-4, CORonavirus MOnitoRing Study parts 3 and 4.

The target population was (a) adults (aged ≥ 18 years), (b) infected by SARS-CoV-2 during the first pandemic wave and who had accessed the Infectious Diseases Unit services of a large academic hospital in the Friuli Venezia Giulia Region (Italy), (c) contactable 24 months post-onset, and (d) who were willing to participate. The exclusion criteria varied according to the stage of the CORMOR3-4 study. As indicated in Figure 1, in the enrollment phase, patients not able to give their consent and those who refused to participate were excluded. At the 24-months follow-up, those who did not suffer from post-COVID-19 syndrome and who did not answer three phone calls (one/week) were all excluded. Overall, a total of 479 patients were invited to participate, and 230 responded at the 24-month follow-up, as reported in Figure 1.

### 2.3. Data Collection Methods and Instruments

Data collection was performed at multiple time points: disease onset, 6 months, 12 and 24 months. At COVID-19 disease onset (a) sociodemographic (e.g., age and gender) and (b) clinical (e.g., COVID-19 severity, hospitalization status [yes/no], length of the in-hospital stays, comorbidities, and symptoms [number and nature]) baseline data were collected. COVID-19 severity was categorized as asymptomatic, mild (without pneumonia), moderate (pneumonia), severe (severe pneumonia), or critical (including acute respiratory distress syndrome—ARDS, sepsis, and/or septic shock [25]). At 6 and 12 months, the same data were collected, and patients were also asked about persistent COVID-19-related symptoms (number) as an indicator of post-COVID-19 syndrome (“yes, I am still suffering”/“no”). At 24 months, data collection was further enriched by asking patients (a) whether they knew the post-COVID-19 syndrome, and the source of their knowledge; (b) whether they were still experiencing post-COVID-19 syndrome as compared to the previous data collection time point (“yes, I am still suffering”/“no”). Those who were suffering from the post-COVID-19 syndrome were requested to describe the syndrome in their own words. This question was open-ended, and participants were free to describe their experience and personal representation of the post-COVID-19 syndrome by giving their own definition, with no assumptions or external influence exerted by the researchers. The interview was allowed to be filled in via telephone or via online platform in the form of questionnaire (EUSurvey) [26]. In the case of telephone interviews, researchers accurately transcribed patients’ responses verbatim.

### 2.4. Data Collection Process and Rigor

Two researchers (a male Registered Nurse [RN] and a female RN, PhD student) conducted telephone interviews from May to November 2022. They had no prior relationship with the interviewees, and they followed a structured protocol for the data collection to ensure reliability of the data collected and neutrality. During the first call, participants were briefly reminded of the study’s aims and asked for their participation consent. Two options for data collection were offered, via phone or online, according to the participant’s personal preferences. In the case of phone interviews, the best moment to perform them was agreed upon and scheduled with participants to ensure calm and prevent disturbances. Patients were given time to reflect and provide accurate answers using their own words without pressure. In the online data collection, the link was sent to the preferred personal email communicated by the participant.

### 2.5. Data Analysis

Qualitative data were analyzed using Graneheim and Lundman’s content analysis approach [20]. Each patient’s description was treated as a single meaning unit because it was “*large enough to be considered a whole and small enough to be possible to keep in mind as a context for the meaning unit*” [20]. Meaning units were grouped by similarities and differences. Codes belonging to the same subject area were further grouped into categories. Finally, similar categories were condensed into themes summarizing patients’ experiences. A corroborative counting technique was applied [27], with a similar approach to previous research experiences on COVID-19 (e.g., [28]), to detect the frequency of the codes and identify the most recurrent. The entire process was performed for all the descriptions expressed in an independent fashion, reaching agreement upon the findings through investigator triangulation [29,30]. Moreover, descriptive analyses were performed for demographic and clinical variables (frequencies, percentages, averages, and confidence intervals [CI] at 95%).

### 2.6. Ethical Issues

Written informed consent was obtained from all participants before the study enrollment. At the 24-month follow-up, participants were re-asked to provide their verbal or written consent. Confidentiality was ensured by anonymizing narratives verbatim transcribed and associating the quotes extracted with the patient’s anonymous identifier number. This study was approved by the Ethics Committee of the Friuli Venezia Giulia Region (Italy), approval numbers CEUR-2020-OS-219 and CEUR-2020-OS-205.

## 3. Results

### 3.1. Patients’ Characteristics

This study included 230 individuals from the initial study cohort (Figure 1), representing 38.4% of the original cohort of 599 participants. Most of them were female (53.5%), aged between 41 and 60 years (43%) and native Italians (89.6%). A significant portion reported at least one comorbidity (53.5%), with hypertension being the most prevalent (20.8%). Participants disclosed their occupational status as either not in contact with the public (31.1%), healthcare workers (22.5%), in occupations involving public interaction (17.7%), or engaged in other types of employment (12%), with a minority being retired (16.8%). A majority experienced mild (67.7%), followed by moderate (24.9%) manifestations of COVID-19, and were generally treated as outpatients (71.3%), with fewer receiving inpatient care (23.5%) or admission to an Intensive Care Unit (5.2%), resulting in an average length of hospital stay of seven days (range 4–10) (Table 1).

Overall, as reported in Table 1, after 24 months, 158 of them (65.8%) reported having experienced post-COVID-19 syndrome, primarily women (59.5%). Most of them had already reported symptoms of post-COVID-19 syndrome at 6 (65.8%) and 12 (72.2%) months. On average, patients experienced three symptoms (range 2–4) and most presented with at least one symptom (52.5%).

### 3.2. Post-COVID-19 Syndrome According to Patients’ Knowledge and Perception

Seventy-five patients reported knowing what the post-COVID-19 syndrome is (47.5%), whilst others did not know (29.1%) or reported being uncertain (23.4%) (Table 2). Out of those who reported some knowledge on the post-COVID-19 syndrome, 45 reported obtaining information from the media (e.g., television, newspapers) (28.9%), others gathered information from the internet (28.2%), their general practitioner (19.2%), and a few from a specialized physician (7.7%). At the 24-months follow up, forty-six patients reported the persistence of post-COVID-19 syndrome (29.1%), whilst others reported not experiencing the syndrome (43.7%) or being uncertain (27.2%).

### 3.3. Post-COVID-19 Syndrome as Described by Patients

A total of 136 patients out of 158 (86.0%) described their experience of post-COVID-19 syndrome using a total number of 1019 words, between 1 and 45 words, an average of 6.4 words per patient. From the qualitative analysis, two main themes emerged (Table 3): (a) experiencing interrelated physical and psychological symptoms and (b) fighting like warriors for a long time.

#### 3.3.1. Experiencing Interrelated Physical and Psychological Symptoms

Patients reported their experience as a “*spectrum of manifestations*” of interrelated physical and psychological symptoms, describing it as a “*set of disorders/diseases that were not there before*” (Table 3). Three categories emerged from the analysis: (a) physical manifestations: struggling between pain and fatigue, (b) emotional storm, and (c) like a fog in my brain.

Regarding the “*Physical manifestations: struggling between pain and fatigue*” (56.6%), patients reported a constellation of physical symptoms, most commonly fatigue (53.2%), followed by physical pain (14.3%) and dyspnea (11.7%). One patient described this experience as a “*bad flu*”. Weakness, lack of appetite, hypoesthesia, sore throat, cough, and weight loss were also described (1.3%, respectively).

Some patients described their experience as more characterized by psychological distress, reporting it as an “*Emotional storm*” of psychologically painful experiences (22.1%) (e.g., “*I see more the psychological side*”), reporting fear (16.7%), anxiety (13.3%), depression (13.3%), and stress (10%). The fear of the COVID-19 disease was described as “*impacting*”, “*an obsession*”, and “*traumatizing*”. A feeling of emotional closure was also reported, e.g., “*A set of symptoms. Sadness, melancholy, no desire like before, because we closed ourselves off*”. Patients also described their psychological status as a novel weight and burden to carry, like “*A pain! There are days when it weighs on me, something I didn’t have before*”. Moreover, patients reported experiencing “*Like a fog in my brain*” (7.4%), describing the perception of a memory loss (50%), mental fatigue (20%), and concentration loss (10%). Expressions used by participants were “*mental fog*” and a “*fog in the brain*”.

#### 3.3.2. Fighting like Warriors for a Long Time

The post-COVID-19 syndrome’s experience was characterized by the sensation that “*Symptoms come and go, we get worse, but we are warriors*”. Three main categories emerged from the analysis: (a) it left something in me, (b) experiencing something new, and (c) an endless story.

Patients reported that COVID-19 “*Left something in me*” (13.2%) in terms of long-term consequences (72.2%), not being as before (16.7%) and feeling impacted (11.1%). According to patients, the syndrome is characterized by “*Long-term consequences caused by COVID-19*” and a “*Syndrome that carries consequences due to the disease*”. The experience was “*Something new*” as never lived before (11.8%), and “*An endless story*” (19.9%) mainly characterized by persistent symptoms (81.5%).

## 4. Discussion

The post-COVID-19 syndrome has been documented in a large proportion of patients, more commonly women [31,32,33,34], older individuals [31,35,36], people with pre-existing health conditions [37,38], and those who experienced a more severe acute phase of the illness [31,35]. A complex spectrum of sequalae has been reported in the literature, encompassing both physical and mental symptoms that affect a patient’s ability to function as before [32,39,40,41,42,43,44]. As expected, in the current study, patients’ descriptions fell within these two broad categories, emphasizing the long-lasting consequences of COVID-19, perceived as a detrimental change compared to the past, which could result in a certain degree of disability and poor quality of life.

We have performed a study valuing the perspective of patients. It is worth mentioning that our increasing knowledge of the post-COVID-19 syndrome is largely due to patient-led initiatives, with initial discussions of lived experiences on social media. Already in 2020, a patient-led survey showed for the first time that COVID-19-related symptoms may persist beyond the acute phase [45]. In 2021, a patient-led research team published the first report describing this emerging syndrome across an international cohort followed up for 7 months [43]. Notably, patients were also primarily involved in advancing policies for those suffering from prolonged symptoms attributed to COVID-19, contributing to the development of research-informed approaches to post-COVID-19 syndrome rehabilitation [46,47].

Recognizing the importance of assessing patients beyond the acute phase, our research group conducted follow-up evaluations over the months and years following the initial COVID-19 outbreak. We believed that some patients might not achieve full recovery (restitutio ad integrum) and could continue to experience long-term malaise [10,11]. Follow-up visits helped map the phenomenon, identifying symptom progression patterns [10]. Specifically, post-COVID-19 symptoms were identified at each follow-up assessment, conducted at 6, 12, and 24 months after onset. One of the most relevant findings was that while some physical symptoms persist, albeit to a lesser extent than during the acute phase, new symptoms also emerge, particularly in the mental health domain, including fatigue and cognitive failures. These symptoms appeared to reach a possible plateau 24 months post-infection, as documented by formal psychometric assessments conducted during follow-up assessment.

Patients’ perspectives have become increasingly central to our study, with efforts made to highlight their lived experiences and emotional processing [48]. Notably, the large majority of COVID-19 survivors (86%) provided at least a few words regarding their post-COVID-19 syndrome (averaging 6.4), with some offering longer descriptions (up to 45 words). Overall, our findings complement previous reports, reaching similar conclusions regarding the impact of the post-COVID-19 syndrome on functioning, disability, and health, but from a patient-centered perspective. Along with pain, fatigue and dyspnea were the most frequently reported symptoms by our participants. This is totally in line with evidence from the Living Systematic Review database [49] and other earlier [50] and more recent reports [51]. While some themes were commonly reported, helping define the syndrome’s primary characteristics (i.e., pain, fatigue, and dyspnea), other, less frequently mentioned symptoms (e.g., weight loss, apathy) underscored the variability of post-COVID-19 manifestations. The richness of the patients’ descriptions highlighted the complexity of this phenomenon, not only with respect to its multifaced clinical presentation, but also to its unique and unprecedented nature.

As previously mentioned, the post-COVID-19 syndrome also led to a broader sense of poor psychosocial functioning, negative emotions [50], and traumatic experiences [51]. This finding is in line with experimental evidence that infections, particularly those causing inflammatory perturbations in the brain [52], may result in sickness behavior symptoms. This group of disturbances includes mood fluctuations, concentration difficulties, reduced social behavior, and diminished responsiveness to social stimuli [53]. This perception of social disability was particularly pronounced for some patients, possibly indicating an inflammation-mediated susceptibility to neuropsychiatric symptoms [54]. This aligns with previous evidence that care for people with the post-COVID-19 syndrome should focus on ensuring that patients feel safe and empowered [55].

Like previous studies, patients’ responses in our cohort reflected their perception that clinicians had limited knowledge of the post-COVID-19 syndrome [56], leading many of them to rely on the internet and social media for information. Many patients admitted they were unfamiliar with the concept of the post-COVID-19 syndrome. Recent updates indicate that while multiple interacting biological mechanisms have been identified, most current clinical approaches to the post-COVID-19 syndrome remain largely symptomatic and supportive, rather than being grounded in these biological mechanisms [57]. Given the widespread prevalence of this condition worldwide—described as a “*mass disabling event*” [58]—further research is needed to close this gap.

This study has several limitations. The absence of a control group of non-COVID-19 patients prevents from quantifying a potential distress attributable to the pandemic itself and its psychosocial consequences, rather than a direct effect of the infection. In other words, we cannot rule out a “psychological post-COVID syndrome”, where depressive symptoms do not result from a biological insult of the infection but from broader psychological distress [59,60]. In addition, the absence of consideration regarding the familial medical history of the patients may affect comprehending the distress levels reported as potentially associated with COVID-19. The mental health burden associated with the post-COVID-19 syndrome is particularly worthy of further investigation. Some studies have found no greater mental distress among COVID-19 survivors compared to individuals hospitalized for other infections [61]. A control group of patients with other medical conditions would help clarify whether COVID-19 induces specific mental health disruptions. Furthermore, no biological assessment was performed that could have provided potential biomarkers of the post-COVID-19 syndrome [62,63]. An identified methodological limitation of our study pertains to the sequencing of the question eliciting thoughts on the post-COVID-19 syndrome, which was positioned before the solicitation of the open-ended question regarding the post-COVID-19 syndrome perspective, potentially influencing the narratives of participants. Additionally, the utilization of mixed administration modes (via phone and online), despite the methodological aspects embodied to ensure rigor, introduced potential variances concerning questionnaire visibility, temporal constraints, and opportunities for reflective engagement. Moreover, this research was not intended to redefine the definition of the post-COVID-19 syndrome as established by medical authorities. Instead, it highlighted the perspectives and lived experiences of patients within the study cohort.

## 5. Conclusions

This study was aimed at investigating the emic perspective of the post-COVID-19 syndrome among a cohort of COVID-19 survivors of the first pandemic wave, two years after the infection onset. Results indicate that the post-COVID-19 syndrome is highly prevalent at 24 months but poorly understood and that patients often rely on low-quality information rather than clinicians. Moreover, the patients’ report is of a complex syndrome encompassing physical, mental, and disability symptoms of an unclear trajectory, with the subjective perception of a set of interrelated physical and psychological symptoms, an experience of struggle between pain and fatigue, an emotional storm, and brain fog perception.

Findings have important clinical implications that may inform policy decisions. First, they suggest the need to focus more on the long-lasting consequences of COVID-19, incorporating insights from individuals with lived experiences of this still partially unknown condition; second, individualized health care services and consultations are needed to support the multidimensional frailty developed by these patients in order to promote their recovery.

## Figures and Tables

**Table 1:** Descriptive characteristics of patients involved at 24 months: overall (*N* = 230) and those reporting as having experienced a post-COVID-19 syndrome.

Patients’ Characteristics	Responded to Follow-Up at 24 Months *N* = 230	Patients Who Experienced Post-COVID-19 Syndrome *N* = 158
Gender, n (%)		
Female	123 (53.5)	94 (59.5)
Male	107 (46.5)	64 (40.5)
Age group, n (%)		
18–40	44 (19.1)	22 (13.9)
41–60	99 (43.0)	76 (48.1)
>60	87 (37.8)	60 (38.0)
Ethnicity, n/N (%)		
Native Italian	206/217 (94.9)	145/152 (95.4)
European	11/217 (5.1)	7/152 (4.6)
Smoking habit, n/N (%)		
Non-smoker	144/228 (63.2)	102/157 (65.0)
Smoker	28/268 (12.3)	17/157 (10.8)
Ex-smoker	56/268 (24.6)	38/157 (24.2)
Alcohol habit, n/N (%)		
Non-drinker	109/227 (48.0)	81/156 (51.9)
Drinker	116/227 (51.1)	74/156 (47.4)
Alcohol use disorder	2/227 (0.9)	1/156 (0.6)
Work, n/N (%)		
HCWs	47 (22.5)	29/146 (19.9)
Work in contact with public	37 (17.7)	26/146 (17.8)
Work not in contact with the public	65 (31.1)	43/146 (29.5)
Retired	35 (16.8)	24/146 (16.4)
Other	23 (12.0)	24/146 (16.4)
Co-morbidities, number, n (%)		
0	107 (46.5)	64 (40.5)
1	66 (28.7)	49 (31.0)
2	32 (13.9)	25 (15.8)
3	16 (7.0)	12 (7.6)
≥4	9 (3.9)	8 (5.1)
Co-morbidities, n/N (%)		
Hypertension	47/226 (20.8)	35/156 (22.4)
Obesity	29 (12.6)	20 (12.7)
Diabetes	15/229 (6.6)	9 (5.7)
Chronic respiratory disease	8/229 (3.5)	8 (5.1)
Cardiovascular disease	4/225 (1.8)	2 (1.3)
Liver disease	7/229 (3.1)	7 (4.4)
Psychiatric disorders	3 (1.3)	2 (1.3)
Renal impairment	0 (0)	0 (0)
Under chronic medication, n/N (%)		
Yes	105/227 (46.3)	78/157 (49.7)
No	122/227 (53.7)	79/157 (50.3)
Acute COVID-19 severity, n/N (%)		
Asymptomatic	17/229 (7.4)	5 (3.2)
Mild	155/229 (67.7)	110 (69.6)
Moderate, Severe and critical	57/229 (24.9)	43 (27.2)
Symptoms at onset, number, n (%)		
0	27 (11.7)	9 (5.7)
1	35 (15.2)	17 (10.8)
2	49 (21.3)	29 (18.3)
3	31 (13.5)	24 (15.2)
4	43 (18.7)	36 (22.8)
≥5	43 (19.6)	43 (27.2)
Management at the onset, n (%)		
Outpatients	164 (71.3)	106 (67.1)
Inpatients		
Ward	54 (23.5)	44 (27.8)
ICU	12 (5.2)	8 (5.1)
Length of in-hospital stay, days, median (IQR)	7 (4–10)	7 (4–9)
Post-COVID-19 syndrome 6 months, n (%)	104 (45.2)	104 (65.8)
Post-COVID-19 syndrome 12 months, n (%)	114 (49.6)	114 (72.2)
At least one post-COVID-19 symptom at 24 months, n (%)	83 (36.1)	83 (52.5)
Number of post-COVID-19 symptoms at 24 months, median (IQR)	3 (2–4)	3 (2–4)
Patients without post-COVID-19 syndrome at 6, 12, 24 months, n/N (%)	72/230 (31.3)	

Legend. N, Sample; n, Number; HCWs, Health Care Workers; COVID-19, Coronavirus Disease 2019; ICU, Intensive Care Unit; IQR, Interquartile Range.

**Table 2:** Post-COVID-19 syndrome according to the knowledge and perception of patients.

At 24 Months After the COVID-19 Onset	*N* = 158
Do you know what is the post-COVID-19 syndrome?, n (%)	
Yes	75 (47.5)
No	46 (29.1)
Maybe/uncertain	37 (23.4)
What is the source of your knowledge regarding the syndrome?, n/N (%)	
General Practitioner	30/156 (19.2)
Specialized Physician	12/156 (7.7)
Internet	44/156 (28.2)
Media (television, newspapers)	45/156 (28.9)
Other	25/156 (16.0)
Do you think you suffer from this syndrome at 24 months?, n (%)	
Yes	46 (29.1)
No	69 (43.7)
Maybe/uncertain	43 (27.2)

Legend. N, Sample; n, Number; COVID-19, Coronavirus Disease 2019.

**Table 3:** Themes, categories, and codes.

Theme	Category	*N* = 136 (100%)
	Codes	
*Experiencing interrelated physical and psychological symptoms*	*Physical manifestations: struggling between pain and fatigue*	77 (56.6)
	Fatigue	41 (53.2)
	Pain	11 (14.3)
	Dyspnea	9 (11.7)
	Alteration of the senses	4 (5.2)
	Faintness	2 (2.6)
	Heart disease	2 (2.6)
	Intestinal disorder	1 (1.3)
	Flu	1 (1.3)
	Weakness	1 (1.3)
	Lack of appetite	1 (1.3)
	Hypoesthesia	1 (1.3)
	Sore throat	1 (1.3)
	Cough	1 (1.3)
	Weight loss	1 (1.3)
	*Emotional storm*	30 (22.1)
	Fear	5 (16.7)
	Anxiety	4 (13.3)
	Depression	4 (13.3)
	Stress	3 (10.0)
	Nuisance	2 (6.7)
	Insomnia	2 (6.7)
	Burden	2 (6.7)
	Trauma	1 (3.3)
	Sadness	1 (3.3)
	Melancholy	1 (3.3)
	Lack of desire	1 (3.3)
	Exhaustion	1 (3.3)
	Concerns	1 (3.3)
	Irritability	1 (3.3)
	Being apathy	1 (3.3)
	*Like a fog in my brain*	10 (7.4)
	Memory loss	5 (50.0)
	Feeling mental fatigue	2 (20.0)
	Being in a mental fog	2 (20.0)
	Losing concentration	1 (10.0)
*Fighting like warriors for a long time*	*It left something in me*	18 (13.2)
	Long-term consequences	13 (72.2)
	I am not as before	3 (16.7)
	Impact	2 (11.1)
	*Experiencing something new*	16 (11.8)
	*An endless story*	27 (19.9)
	Persistent symptoms	22 (81.5)
	Debilitating syndrome	4 (14.8)
	Intermittent symptoms	1 (3.7)

Legend. N, Sample.

## Data Availability

The data presented in this study are available on reasonable request from the corresponding author due to privacy concerns.

## Supplementary Materials

The following supporting information can be downloaded at: https://www.mdpi.com/article/10.3390/healthcare13070757/s1; Table S1: Post-COVID-19 syndrome’s names and definitions provided by health authorities; Table S2: Standards for Reporting Qualitative Research (SRQR) checklist [24,64,65,66,67,68,69,70].

## Abbreviations

The following abbreviations are used in this manuscript:

ARDS	Acute Respiratory Distress Syndrome
COVID-19	Coronavirus Disease 2019
CORMOR	CORonavirus MOnitoRing
HCW	Healthcare Worker
PHEIC	Public Health Emergency of International Concern
P	Patient’s anonymous identifier number
PhD	Doctor of Philosophy
RN	Registered Nurse
SARS-CoV-2	Severe Respiratory Syndrome Coronavirus 2
SRQR	Standards for Reporting Qualitative Research
WHO	World Health Organization
