# Supplementary material for: Post-COVID-19 Syndrome as Described by Patients: A Qualitative Study
Source: Healthcare (Basel). 2025 Mar 28;13(7):757. doi: 10.3390/healthcare13070757 (PMC11989186; doi:10.3390/healthcare13070757)
Supplement: Supplementary file 1 [file healthcare-13-00757-s001.zip › healthcare-3502988-supplementary.pdf]

Supplementary Table S1. Post-COVID-19 syndrome's names and definitions provided by health authorities.

Source	Name(s) attributed	Definition	Date and reference^
World Health Organization (WHO)	Post COVID-19 Condition	Post COVID-19 condition occurs in individuals with a history of probable or confirmed SARS CoV-2 infection, usually 3 months from the onset of COVID-19 with symptoms and that last for at least 2 months and cannot be explained by an alternative diagnosis. Common symptoms include fatigue, shortness of breath, cognitive dysfunction but also others and generally have an impact on everyday functioning. Symptoms may be new onset following initial recovery from an acute COVID-19 episode or persist from the initial illness. Symptoms may also fluctuate or relapse over time.	Oct 2021 [1]
	Post COVID-19 Condition "Long COVID"	Post COVID-19 condition, also known as long COVID, refers to long-term symptoms that some people experience after they have had COVID-19. People who experience post COVID-19 condition sometimes refer to themselves as "long-haulers". These symptoms might persist from their initial illness or develop after their recovery. They can come and go or relapse over time. The most common symptoms associated with post COVID-19 condition include fatigue, breathlessness and cognitive dysfunction (for example, confusion, forgetfulness, or a lack of mental focus or clarity). Post COVID-19 condition can affect a person's ability to perform daily activities such as work or household chores.	Mar 2023 [2]
World Health Organization (WHO) Europe	Post COVID-19 Condition (commonly known as) Long COVID	It is defined as the continuation or development of new symptoms 3 months after the initial SARS-CoV-2 infection, with these symptoms lasting for at least 2 months with no other explanation.	Dec 2022 [3]
Centers for Disease Control and Prevention (CDC) United States of America	Post-COVID Conditions<br><br>Other names: Long COVID, long-haul COVID, post-acute COVID-19, post-acute sequelae of SARS CoV-2 infection (PASC), long-term effects of COVID, and chronic COVID.	Long COVID is broadly defined as signs, symptoms, and conditions that continue or develop after initial COVID-19 or SARS-CoV-2 infection. The signs, symptoms, and conditions are present four weeks or more after the initial phase of infection; may be multisystemic; and may present with a relapsing– remitting pattern and progression or worsening over time, with the possibility of severe and life-threatening events even months or years after infection. Long COVID is not one condition. It represents many potentially overlapping entities, likely with different biological causes and different sets of risk factors and outcomes.	Aug 2022 [4]
National Health Service (NHS) United Kingdom	Long COVID<br>Post COVID-19 syndrome	Most people with coronavirus (COVID-19) feel better within a few days or weeks of their first symptoms and make a full recovery within 12 weeks. For some people, symptoms can last longer. This is called long COVID or post COVID-19 syndrome. Long COVID is a new condition which is still being studied.	Mar 2023 [5]

National Institute for Health and Care Excellence (NICE) United Kingdom	Post-COVID-19 syndrome<br>Long COVID	Signs and symptoms that develop during or after an infection consistent with COVID-19, continue for more than 12 weeks and are not explained by an alternative diagnosis. It usually presents with clusters of symptoms, often overlapping, which can fluctuate and change over time and can affect any system in the body. Post-COVID-19 syndrome may be considered before 12 weeks while the possibility of an alternative underlying disease is also being assessed. In addition to the clinical case definitions, the term 'long COVID' is commonly used to describe signs and symptoms that continue or develop after acute COVID-19. It includes both ongoing symptomatic COVID-19 (from 4 to 12 weeks) and post-COVID-19 syndrome (12 weeks or more).	Jan 2024 [6]
Istituto Superiore di Sanità (ISS) Italy	Long-COVID<br>Post-COVID-19 sydrome<br>Symptomatic persistent COVID-19 disease	Symptomatic persistent COVID-19 disease: signs and symptoms attributable to COVID-19 lasting between 4 and 12 weeks after the acute event.<br>Post-COVID-19 syndrome: signs and symptoms that developed during or after an infection compatible with COVID-19 present for more than 12 weeks after the acute event and cannot be explained by alternative diagnoses.<br>Long-COVID includes both the persistent symptomatic form and the post-COVID syndrome.<br>This condition is therefore characterized by signs and symptoms caused by SARS-CoV-2 infection that continue or develop 4 weeks after an acute infection. The use of shared terminologies provides the basis for service planning, facilitates care and enables the definition of a clinical dataset necessary for monitoring and research.	Jul 2021 [7]

Legend: COVID-19, Coronavirus Disease 2019; COVID, Coronavirus Disease; WHO, World Health Organization; PASC, post-acute sequelae of Severe Acute Respiratory Syndrome Coronavirus 2 infection; SARS-CoV-2, Severe Acute Respiratory Syndrome Coronavirus 2; CDC, Centers for Disease Control and Prevention; NHS, National Health Service; NICE, National Institute for Health and Care Excellence; ISS, Institute Superiore di Sanità.

^ For definitions published on web pages, the date reported is the date of the last update of the web page.

Supplementary Table S2. Standards for Reporting Qualitative Research (SRQR) checklist [8].

No.	Topic	Item	Location reported
S1	Title	Concise description of the nature and topic of the study Identifying the study as qualitative or indicating the approach (e.g., ethnography, grounded theory) or data collection methods (e.g., interview, focus group) is recommended.	Title
S2	Abstract	Summary of key elements of the study using the abstract format of the intended publication; typically includes background, purpose, methods, results, and conclusions.	Abstract
S3	Problem formulation	Description and significance of the problem/phenomenon studied; review of relevant theory and empirical work; problem statement.	The problem formulation is reported in the Introduction section.
S4	Purpose or research question	Purpose of the study and specific objectives or questions.	The research question/ purpose is reported in the Introduction section.
S5	Qualitative approach and research paradigm	Qualitative approach (e.g., ethnography, grounded theory, case study, phenomenology, narrative research) and guiding theory if appropriate; identifying the research paradigm (e.g., postpositivist, constructivist/interpretivist) is also recommended; rationale.	A descriptive qualitative approach was used. The rationale is reported in the Methods section.
S6	Researcher characteristics and reflexivity	Researchers' characteristics that may influence the research, including personal attributes, qualifications/experience, relationship with participants, assumptions, and/or presuppositions; potential or actual interaction between researchers' characteristics and the research questions, approach, methods, results, and/or transferability.	Researchers were nurses educated at the advanced level, with previous experience in qualitative studies, with no assumptions/presuppositions and with no previous relationship with the patients.
S7	Context	Setting/site and salient contextual factors; rationale.	An Infectious Diseases Unit, reference centre for COVID-19 patients located in the northeast of Italy. This is a qualitative descriptive study nested in a large prospective monocentric cohort study named COROnavirus MOnitoRing part 4.
S8	Sampling strategy	How and why research participants, documents, or events were selected; criteria for deciding when no further sampling was necessary (e.g., sampling saturation); rationale.	The target population is described in Methods. In Figure 1, the patients' flow chart is summarized.
S9	Ethical issues pertaining to human subjects	Documentation of approval by an appropriate ethics review board and participant consent, or explanation for lack thereof; other confidentiality and data security issues.	Ethical considerations are described in the Method section.
S10	Data collection methods	Types of data collected; details of data collection procedures including (as appropriate) start and stop dates of data collection and analysis, iterative process, triangulation of sources/methods, and modification of procedures in response to evolving study findings; rationale.	Data collection process and rigor are summarized in the Method section. Data collection instruments and technologies are summarized in the Method section.
S11	Data collection instruments and technologies	Description of instruments (e.g., interview guides, questionnaires) and devices (e.g., audio recorders) used for data collection; if/how the instrument(s) changed over the course of the study.	Data are summarized in Table 1, in Supplementary Table 2, and in the Results section.
S12	Units of study	Number and relevant characteristics of participants, documents, or events included in the study; level of participation (could be reported in results).	Data processing is summarized in the Method section.
S13	Data processing	Methods for processing data prior to and during analysis, including transcription, data entry, data management and security, verification of data integrity, data coding, and anonymization/deidentification of excerpts.	Data processing is summarized in the Method section.

S14	Data analysis	Process by which inferences, themes, etc., were identified and developed, including the researchers involved in data analysis; usually references a specific paradigm or approach; rationale.	Data analysis is summarized in Method section.
S15	Techniques to enhance trustworthiness	Techniques to enhance trustworthiness and credibility of data analysis (e.g., member checking, audit trail, triangulation); rationale.	Data collection process and rigor are summarized in Method section.
S16	Synthesis and interpretation	Main findings (e.g., interpretations, inferences, and themes); might include development of a theory or model, or integration with prior research or theory.	The main findings are reported in Results section.
S17	Links to empirical data	Evidence (e.g., quotes, field notes, text excerpts, photographs) to substantiate analytic findings.	Evidence to substantiate analytic findings is reported in Results section.
S18	Integration with prior work, implications, transferability, and contribution(s) to the field	Short summary of main findings; explanation of how findings and conclusions connect to, support, elaborate on, or challenge conclusions of earlier scholarship; discussion of scope of application/generalizability; identification of unique contribution(s) to scholarship in a discipline or field.	The discussion of the findings considering previous work has been summarized in Discussion section.
S19	Limitations	Trustworthiness and limitations of findings.	The main limitations are presented at the end of Discussion section. Strategies preventing some limitations have been reported in Methods section.
S20	Conflicts of interest	Potential sources of influence or perceived influence on study conduct and conclusions; how these were managed.	None.
S21	Funding	Sources of funding and other support; role of funders in data collection, interpretation, and reporting.	None.

References

1. World Health Organization. A clinical case definition of post COVID-19 condition by a Delphi consensus. Available online: https://www.who.int/publications/i/item/WHO-2019-nCoV-Post_COVID-19_condition-Clinical_case_definition-2021.1 (accessed on 17/01/2025).
2. World Health Organization. Coronavirus disease (COVID-19): Post COVID-19 condition. Available online: https://www.who.int/news-room/questions-and-answers/item/coronavirus-disease-(covid-19)-post-covid-19-condition (accessed on 17/01/2025).
3. World Health Organization Europe. Post COVID-19 condition (Long COVID). Available online: https://www.who.int/europe/news-room/fact-sheets/item/post-covid-19-condition (accessed on 14/01/2025).
4. Centers for Disease Control and Prevention. National Research Action Plan on Long COVID. Available online: https://www.covid.gov/sites/default/files/documents/National-Research-Action-Plan-on-Long-COVID-08012022.pdf (accessed on 17/01/2025).
5. National Health Service. Long-term effects of COVID-19 (long COVID). Available online: https://www.nhs.uk/conditions/covid-19/long-term-effects-of-covid-19-long-covid/ (accessed on 14/01/2025).
6. National Institute for Health and Care Excellence. COVID-19 rapid guideline: managing the long-term effects of COVID-19. Available online: https://www.nice.org.uk/guidance/ng188/chapter/1-Identification (accessed on 17/01/2025).
7. Onder, G.; Floridia, M.; Giuliano, M.; Lo Noce, C.; Tiple, D.; Bertinato, L.; Mariniello, R.; Laganà, M.G.; Della Vecchia, A.; Gianferro, R.; et al. *Indicazioni ad interim sui principi di gestione del Long-COVID*; Istituto Superiore di Sanità: Roma, 2021-07-01 2021.
8. O'Brien, B.C.; Harris, I.B.; Beckman, T.J.; Reed, D.A.; Cook, D.A. Standards for reporting qualitative research: a synthesis of recommendations. *Acad. Med.* **2014**, *89*, 1245-1251, doi:10.1097/acm.0000000000000388.
